# Possibility of long-term survival of African swine fever virus in natural conditions

**DOI:** 10.14202/vetworld.2021.854-859

**Published:** 2021-04-09

**Authors:** Hranush Arzumanyan, Sona Hakobyan, Hranush Avagyan, Roza Izmailyan, Narek Nersisyan, Zaven Karalyan

**Affiliations:** 1 Laboratory of Cell Biology and Virology, Institute of Molecular Biology of NAS RA, Yerevan, Armenia; 2 Experimental Laboratory, Yerevan State Medical University, Yerevan, Armenia

**Keywords:** African swine fever virus, p72 gene, virus infectivity, virus survival

## Abstract

**Background and Aim:**

In modern scientific literature presents an understanding that African swine fever (ASF) ASF virus (ASFV) is remarkably stable in the environment, and carcasses of the pigs which were died after ASF, play a key role as ASFV reservoir. The aim of this study was to evaluate the possibility of the ASFV (different isolates) survival in bodies of dead animals, bones, remnants of bone marrow, residual organ matrix in natural conditions.

**Materials and Methods:**

Skeletons of ASFV infected pigs which were died and left/abandoned in forests or buried in Armenia at diverse time points and locations had been excavated and examined for the presence of ASFV genome by real-time polymerase chain reaction (PCR) assay and for infection abilities through *in vitro* (hemadsorption test and infection in porcine lung macrophages) as well as by intramuscular infection in healthy pigs.

**Results:**

Current exploration showed that in several samples (with different times of exposure) of excavated skeletons had been detected the presence of the virus gene (p72) using real-time PCR. However, in none of these porcine samples, infectious ASFV could be isolated. Data obtained by real-time PCR at frequent intervals indicated the presence of the virus gene (p72), especially within the case of the acute form of the disease. This can be explained by the highest levels of the virus during the latter case mentioned above.

**Conclusion:**

ASFV seems to be very sensitive to environmental temperature. The best place for ASFV long-term survival in the natural environment is bone marrow from intact big tubular bones (like femur or tibia) of buried carcasses. In artificial “graves,” complete bones with not destructed bone marrow can preserve the virus gene (p72) for a very long time (more than 2 years). Infectious particles in underground conditions survive not so long: In complete bones with not affected bone marrow, possible presence of the virus for several months.

## Introduction

At present, the ability of the African swine fever (ASF) ASF virus (ASVF) to circulate for a long time in the natural conditions in Europe and Asia has been shown. Furthermore, it was also reported on the frequency of new outbreaks of the ASFV in the Eurasian region [1]. In the African region, ASFV can persist in various Suidae susceptible to the virus, and this is not common in Eurasian region. As we know, for the majority of the viruses, including ASFV, there are two main transmission pathways: Direct (infected and susceptible Suidae) and indirect (between Suidae and virus present in infected carcasses or soil (etc.) or carried by any forms of vectors or reservoirs) [2-4]. Lately, it has emerged that prolonged circulation of the ASFV in the wild leads to the appearance of weakened variants of the virus, causing chronic or even asymptomatic infection. Clinical ASF symptoms are more varied and often difficult to distinguish in diseases caused by a low-virulent virus. The causative agent of ASF can remain in infected animals for several months without any obvious clinical signs [1,5]. Furthermore, the long-term circulation of the ASFV in nature is related to the uniqueness of its biological properties and its long-term preservation in the environment [6-9]. Those properties are based on the complex structural organization of the virion.

Nowadays, it has become clear that the ASFV can be transmitted directly during contact between infected and susceptible pigs, by consumption of the meat from infected pigs, through the bites of infected acari (*Ornithodoros* spp.), and through contact with virus contaminated materials or objects (bedding, feed, equipment, clothes and footwear, and vehicles) and fluids such as blood, feces, urine, or saliva from infected pigs [10].

The methods of combating ASF these days include the eradication of sick animals or separation of healthy and considered to be in contact animals, as well as within the imposition of quarantine on an area with a radius of up to 15 kilometers around the place of the established focus. However, the elimination of ASF outbreaks depends now not only on the extent of biosecurity and the effectiveness of the moves of the veterinary service but also on the presence of vectors of transmission and elements of spreading out of the pathogen. Hence, it became clear the importance of investigations of the virus survival in natural environment.

The aim of this study was to evaluate the possibility of the ASFV (different isolates) survival in bodies of dead animals, bones, remnants of bone marrow, residual organ matrix in natural conditions.

## Materials and Methods

### Ethical approval

Animal care and euthanasia were done according to the AVMA Guidelines on Euthanasia and local guidelines for animal care and use (Institutional Review Board/Independent Ethics Committee of the Institute of Molecular Biology of NAS, IRB00004079).

### Sample collection

Samples were collected from the forests of Tavush region. The collection of blood and internal organ samples was begun in 2009. The bones of pig skeletons and parts of carcasses have been collected since 2010. Virological studies were carried out immediately after the collection of samples from the environment. The samples of contaminated soil and feces were obtained after experimental infections in 2015, 2017, and 2018. Samples processing was performed in Laboratory of Cell Biology and Virology, Institute of Molecular Biology of NAS RA, Yerevan, Armenia.

### Animals

Carbon dioxide inhalation (75-80% carbon dioxide for 20 min) was used to euthanatize animals. Experimental infection was carried out on 4-month-old pigs (n=15) by intramuscular injection of extract from collected samples.

### Virus

Virus titration was performed as described previously and expressed as log 10 of hemadsorption dose (HAD) 50/mL for non-adapted cells [11]. ASFV was obtained from spleens originating from infected pigs. The acute form of ASFV (Arm07) was first isolated in 2007 from the spleen of a swine infected with ASFV [12]. The virus belonged to genotype II, which was distributed in Trans-Caucasian countries. The dose of the virus in the spleen (before freezing) extracts was 5.5-6.0 lg HADU 50/mL, in the blood (before freezing) 4.5-5 lg HADU 50/mL. The chronic form of ASFV (Dilijan2011IMB) was first isolated in 2011 from the spleen of swine [5]. The dose of the virus in the spleen (before freezing) extracts was 4-4.5 lg HADU 50/mL, in the blood (before freezing) 2-3.5 lg HADU 50/mL.

### Isolation of DNA and implementation of real-time PCR

Nucleic acid extraction from all examined samples (volume of 0.2 mL) was performed according to the manufacturer’s instructions through Viral Genomic DNA/RNA Extraction Kit (MyBioSource, San Diego, California, USA; catalog number MBS846571). In our samples, the presence of ASFV becomes measured by using a quantitative actual-time polymerase chain reaction with a Rotor Gene Q instrument (Qiagen, USA). DNA of 2 μl (corresponding to 40 ng) was added to 18 μl PCR mix. The very last reaction mix contained 500 nM primers and 250 nM probe (PrimeTime qPCR Assay, IDT, USA), 10 mM MgCl_2_, 200 μM each dNTPs, 1 U Hot-start Taq polymerase, and 1×PCR Buffer (Dia-M, Moscow, Russia). The primers and fluorescent-labeled probe has been used as follows:

#### ASFV gene

Fluorescent probe – 6-FAM/TAMRA.
Sequence 1 – TGC TCA TGG TAT CAA TCT TAT CG.
Sequence 2 – CCA CTG GGT TGG TAT TCC TC.
Sequence 3 – /56-FAM/TTC CAT CAA AGT TCT GCA GCT CTT/36-TAMSp/.

#### β-actin gene

Fluorescent probe – TET/ZEN/IBFQ.
Sequence 1 – CTC GAT CAT GAA GTG CGA CGT.
Sequence 2 – GTG ATC TCC TTC TGC ATC CTG TC.
Sequence 3 – /5TET/AT CAG GAA G/Zen/G ACC TCT ACG CCA ACA CGG/3IABkFQ/.

The β-actin gene has been consumed as a housekeeping gene.

### Porcine alveolar macrophage (PAM) culture

Three-month-old pigs had been euthanized, and the lungs had been removed. Cells obtained during bronchoalveolar lavage were resuspended in sterile Hank’s balanced salt solution. They were centrifuged at 600 g for 10 min and resuspended in Roswell Park Memorial Institute (RPMI) 1640 with 5% fetal bovine serum (FBS) at a cell concentration of 0.5-1×10^6^ cells per mL. After 3 h at 37°C in a humidified, CO_2_ incubator, the adhered cells were washed 3 times with RPMI to remove contaminating non-adherent cells and then reincubated in RPMI 1640 with 10% FBS [13].

The infection of PAM was performed by maximal dilutions of all investigated samples (1×10-1 HADU 50/mL logarithmic dilutions). Before the infection, the experimental samples were tested by HADU and real-time PCR methods for the detection of the ASFV. All samples with PAM were assessed for viability by trypan blue exclusion test. The viability of cells before and after investigations was at least 95-97%.

### Statistical analysis

All *in vitro* experiments have been conducted in triplicate. The significance of virus-triggered modifications has been evaluated through a two-tailed Student’s t-test; p<0.05 had been considered sizeable. SPSS version 17.0 (SPSS Inc., Chicago, IL, USA) has been applied for statistical analyses.

## Results

### Sample collection

In total, more than 70 bone and bone marrow samples and 30 tissue samples from skeletons and carcasses obtained from forests and excavated from cemeteries at 17 different locations had been acquired. Freeze samples had been taken from laboratory collection. The main part of these samples is presented in Tables-1 and 2.

**Table-1:** Surviving of ASFV in bones, remnants of bone marrow, and feces of infected pigs.

S. no.	Type of ASFV	Sample	Time (years)	Real-time PCR	Infection in PAM	HADU	Infection in pig
1.	A1	Spleen ASFV infected pig	1.1	Positive	Positive	Positive	Positive
2.	B1	Spleen-1 healthy pig	-	Negative	Negative	Negative	Not carried out
3.	C1	Spleen-2 healthy pig	-	Negative	Negative	Negative	Not carried out
4.	D1	Blood healthy pig	-	Negative	Negative	Negative	Not carried out
5.	Chronic	Mandible cemetery	5	Negative	Negative	Negative	Not carried out
6.	Chronic	Mandible cemetery	4	Negative	Negative	Negative	Not carried out
7.	Acute	Ribs cemetery	6	Positive	Negative	Negative	Not carried out
8.	Acute	Ribs cemetery	4	Positive	Negative	Negative	Not carried out
9.	Chronic	Femur cemetery	5	Negative	Negative	Negative	Not carried out
10.	Chronic	Femur cemetery	6	Negative	Negative	Negative	Not carried out
11.	Chronic	OS ileus forest	5	Negative	Negative	Negative	Not carried out
12.	Chronic	OS ileus forest	7	Negative	Negative	Negative	Not carried out
13.	Acute	Femur head cemetery	7	Positive	Negative	Negative	Not carried out
14.	Acute	Femur head cemetery	5	Positive	Negative	Negative	Not carried out
15.	Acute	OS femoralis bone marrow cemetery	8	Negative	Negative	Negative	Not carried out
16.	Acute	OS femoralis bone marrow cemetery	7	Positive	Negative	Negative	Not carried out
17.	Chronic	Fibula forest	5	Negative	Negative	Negative	Not carried out
18.	Chronic	Fibula forest	5	Negative	Negative	Negative	Not carried out
19.	Acute	Fibula bone marrow cemetery	8	Negative	Negative	Negative	Not carried out
20.	Acute	Fibula bone marrow cemetery	6	Positive	Negative	Negative	Not carried out
21.	Acute	Fibula bone marrow cemetery	5	Positive	Negative	Negative	Not carried out
22.	Acute	Fibula bone marrow cemetery	8	Negative	Negative	Negative	Not carried out
23.	Chronic	OS femoralis bone marrow forest	4	Positive	Negative	Negative	Not carried out
24.	Chronic	OS femoralis bone marrow forest	5	Positive	Negative	Negative	Not carried out
25.	Acute	Feces forest	2.5	Negative	Negative	Negative	Not carried out
26.	Acute	Feces forest	1.8	Negative	Negative	Negative	Not carried out
27.	Acute	OS femoralis bone marrow cemetery	1.8	Negative	Negative	Negative	Not carried out
28.	Acute	Fibula bone marrow cemetery	1.8	Negative	Negative	Negative	Not carried out
29.	Acute	Mandible cemetery	1.8	Negative	Negative	Negative	Not carried out
30.	Chronic	Mandible cemetery	1.8	Negative	Negative	Negative	Not carried out
31.	Chronic	fibula cemetery	1.8	Positive	Negative	Negative	Not carried out
32.	Acute	OS femoralis bone marrow cemetery	0.8	Positive	Positive*	Negative	Negative
33.	Acute	Fibula bone marrow cemetery	0.8	Positive	Negative	Negative	Negative
34.	Chronic	OS femoralis bone marrow cemetery	0.8	Positive	Negative	Negative	Negative
35.	Chronic	Fibula bone marrow cemetery	0.8	Positive	Negative	Negative	Negative
36.	Acute	Mandible cemetery	0.8	Positive	Negative	Negative	Not carried out
37.	Chronic	Mandible cemetery	0.8	Positive	Negative	Negative	Not carried out
38.	Chronic	Feces	1.5	Negative	Negative	Negative	Negative
39.	Chronic	Feces	2	Negative	Negative	Negative	Not carried out
40.	Acute	Feces	1.5	Negative	Negative	Negative	Negative
41.	Acute	Feces	2	Negative	Negative	Negative	Not carried out

* There is only one positive infection from three cases. ASFV=African swine fever virus, HADU=Hemadsorption units, PAM=Porcine alveolar macrophage, RT-PCR=Reverse transcription–polymerase chain reaction

**Table-2:** Surviving of ASFV in freeze conditions.

S. no.	Type of ASFV	Sample	Time (years)	Real-time PCR	Infection in PAM	HADU	Infection in pig
1.	Acute	Spleen freeze (−28-30°C)	10	Positive	Negative	Negative	Negative
2.	Acute	Spleen freeze (−28-30°C)	7	Positive	Positive	Positive	Positive
3.	Acute	Blood freeze (−28-30°C)	7	Positive	Positive	Positive	Positive
4.	Acute	Blood freeze (−28-30°C)	9	Positive	Positive	Positive	Negative
5.	Chronic	Spleen freeze (−28-30°C)	8	Negative	Negative	Negative	Negative
6.	Chronic	Spleen freeze (−28-30°C)	6	Negative	Negative	Negative	Negative
7.	Chronic	Spleen freeze (−28-30°C)	5	Positive	Positive	Positive	Positive
8.	Chronic	Blood freeze (−28-30°C)	7	Negative	Negative	Negative	Negative
9.	Chronic	Blood freeze (−28-30°C)	7	Negative	Negative	Negative	Negative
10.	Chronic	Blood freeze (−28-30°C)	6	Negative	Negative	Negative	Negative

ASFV=African swine fever virus, HADU=Hemadsorption units, PAM=Porcine alveolar macrophage

### Infectious virus isolation

There was no success of ASFV isolation from all of the investigated samples obtained from excavated skeletons. In the samples, which had been passaged three times on PAMs and in those without previous passaging, were not detected positive HADU reaction and genome could be detected again by real-time PCR.

As follow from Table-1, data obtained by real-time PCR often give the presence of the virus gene (p72), especially in the case of acute form of the disease/ailment. This can be explained by the higher levels of the virus in the case of acute form of ASF [5]. It must be noted that in some samples, which were investigated with real-time PCR, the positive results might be related to the fact that remnants of the tissues were originally preserved in the samples. It is also important to indicate that from three cases we had only one case of positive infection, but HADU test in the same sample was negative, which can be explained by the high sensitivity of real-time PCR method (in genome evaluations) compared with HADU (which detects infection virions).

The presence of the infectious particles of ASFV in almost all cases with positive real-time PCR data has not been confirmed (one excluding PAM infection with the sample from buried skeleton 0.8 years old, bone marrow obtained from OS femoralis – Table-1). There was no successful introduction to the healthy pigs from all investigated samples. Some parts of the ASFV genome have been survived better in underground conditions compared with the surface. For example, feces, which were in a more superficial layer of the earth, and femur or fibula, which were buried deeper. The data about the virus surviving in freezing conditions were obtained since 2009 when our laboratory started working on ASFV and was previously not published. The research results are shown in Table-2. As follow from Table-2 in freezed samples, the virus had been survived at least 5-7 years. At this time point, the virus is preserved its infectivity not only *in vitro* but also *in vivo* conditions. The last data gave us evidence about the amount of the virus particles with infectivity preserved in investigated samples [14].

## Discussion

Long-term survival of the virus in the environment can be dependent on several abilities. First, it is the resistance of the virus particles out of infected cells in contaminated soil, water, biological products, carcasses, stool, etc. It should be noted that despite the influence of many physicochemical factors (temperature, putrefaction, ultrasonic waves, and pH), viral particles retain their stability and can help with the transmission process. Second, it is the presence of the virus in survived animals that may act as carriers of the ASFV, and the possibility of such carries for disease persistence and spread [15]. Two types of carriers or survivors had been examined: 1. Pigs, who developed the chronic type of the disease, and which would ultimately die from ASFV, could become a reason for the resumption of the viremia in the environment. In general, this kind of infection had been related to low virulence. 2. Pigs that were not persistently infected independently of the pathogenicity of the virus. Pigs, which will recover from ASFV thus becoming carriers of the disease, can withstand extended cycles in blood or tissues (including fat and muscle) of the surviving animals. The latter may lead to the transmission of the virus, eventually not only with direct but also with indirect pathways.

Third, it is the possibility of the virus surviving in biological systems, such as arthropod or other invertebrate hosts [16,17]. It should be indicated; these kinds of biological systems are considered as vectors for ASFV. Such vectors (biological, mechanical) are (1) soft ticks of the *Ornithodoros* genus (biological reservoirs for ASFV), which can independently transmit the virus to the susceptible hosts (vertebrate). (2) Hard ticks of the Ixodes genus and Dermacentor genus are known as mechanical vectors of ASFV. (3) Blood-feeding stable fly Stomoxys calcitrans and non-blood-feeding flies (*Culicidae* spp., *Musca domestica*, *Drosophila* spp.) have been reported as mechanical vectors. (4) ASFV infection was also detected in the leech *Hirudo medicinalis* (mechanical vector and possible reservoir).

In general, these vectors can not only be involved in maintainers of the infectious virus but also of possible transmission of the latter. Because of the lack of a sylvatic cycle for the last two cases mentioned above, it is evident that some kind of alternative pathway exists for the maintenance and transmission of ASFV as there is no obliteration of the virus from the environment. Further studies of these vectors maybe will lead to the latter (vectors) being considered as reservoirs for the virus, which will be a source of great achievements from the point of view of ecology and epidemiology.

20^th^-century scientific research has been shown that the ASFV is resistant to survival, including exposure to heat, desiccation, and decay [6-9]. It was found that hams produced from ASFV infected meat were infectious *in vivo* until about 1 year [18]. Several articles described that ASFV could persist for a long time in the carcasses of Suidae [10]. However, we can conclude that there is no strong evidence that the virus can remain infectious in the environment (buried, on the surface of the earth, in feces, in tissue ,or in the ground) for more than 1 year. Moreover, our investigations have been shown that there is a low possibility for infectious virus surviving (at least in the environment of Armenia) in a time period of more than 6 months. Our data also indicated the absence of any successful transmission of ASFV obtained from environmental samples to animals. The possibility of the ASFV to infect a susceptible animal depends on the dose of the virus [19].

The best place for ASFV to survive in the natural environment was bone marrow from intact big tubular bones (such as femur, humerus, or tibia) of buried carcasses. Outside of pig carcasses, in the environment, the survival of the virus was unlikely to be possible over a longer period. Furthermore, in our opinion [20], the real survival time of the infectious viral particle (virion) outside the tissues of pigs probably did not exceed 1 month in the summer (even in the absence of direct exposure to sunlight). In artificial “graves,” complete bones with undamaged bone marrow could preserve the virus gene (p72) for a very long time (more than 2 years). According to our results, infectious particles have been survived not so long in underground conditions: In complete bones with not affected bone marrow was possible the presence of the virus for several months.

It is well known that, in general, the ASFV is highly sensitive to ambient temperature. The latest data on the survival of the virus in forage plants [21] also confirmed conclusions about the impossibility of a long existence of the virus without a sensitive host that could support viral replication and/or long-term survival of the ASFV. Moreover, our data showed that the evolution of the ASFV outside Africa have not led to an increase in the survival time of the virus outside the host, and new virus isolates generally survived less well in the environment than earlier ASFV isolates (Arm07, Georgia07).

It is important to note that free-ranging pigs can easily be considered as a factor responsible for the spread of the virus due to one of the alternative ways of their feeding: Feeding on the carcasses of pigs (also remains of infected pigs) that have died after the disease. This circumstance can also confirm the importance of this study.

Hence, in our opinion related to mechanisms of ASFV’s unique prevalence and stability, it is important to talk about the type of indirect transmission, in which the biological and mechanical vectors have their significant role. For African region, it was reported that soft ticks of the genus *Ornithodoros* spp. can serve as ASFV mechanical vectors. Until recently, there was an absence of any specific vectors for ASFV (*Ornithodoros mubata*), and there are no virus-resistant hosts capable of long-term preservation of the virus without clinical manifestations (wild forest pigs and warthogs). During the last few years, it had been reported about the crucial role of some blood-feeding invertebrates, ticks, leeches, and lice; however, their involvement in the transmission of ASFV had not been studied. Recently, it has been reported about the role of various insects in the indirect transmission of ASFV [3]. However, as shown in scientific data the survival of ASFV in insects is possible only for several days. This way, it is relevant to study the conditions and timing of ASFV survival *in vivo* as these parameters can be the key indices of viral circulation in Eurasian region.

Our data are consistent with the conclusions obtained in the previous study [16], about the importance of obtaining more knowledge related to the survival and transmission of the ASFV through potential intermediate hosts in Eurasia. The possible role of till now unknown hosts which can serve as niches for virus surviving supported by data of the seasonality of the ASF outbreaks in Europe [22]. Knowledge derived from this kind of study can play a crucial role also in undertaking some mechanisms that will limit the spread of the virus.

## Conclusion

Infection with ASFV from bone material and/or soft-tissue remained after being in the environment and/or underground for more than 0.5 years is unlikely. The ASFV can remain infectious for a long time (several years) in an artificial microclimate – when freezing from −20 to −30°C. Isolates Arm07, Georgia07 survive better both in the environment and in an artificial microclimate, compared to the virus that causes the chronic form of the disease (Dilijan 2011IMB). This is probably due to the higher titers of the virus in the tissues and blood of infected pigs in the acute form of ASF compared with the virus causing the chronic form of the disease.

## Authors’ Contributions

HA suggested the topic of the study, collected samples from forests and cemeteries. SH, RI, NN, and HrA performed real-time PCR, HADU measurements, POM investigations, and *in vivo* studies. ZK supervised and wrote the manuscript. All authors read and approved the final manuscript.
